# Does Early Treatment Improve Clinical Outcome of Class II Patients? A Retrospective Study

**DOI:** 10.3390/children9020232

**Published:** 2022-02-09

**Authors:** Roland Männchen, Marco Serafin, Rosamaria Fastuca, Alberto Caprioglio

**Affiliations:** 1 Independent Researcher, 8400 Winterthur, Switzerland; roland.maennchen@gmx.ch; 2 Department of Biomedical Sciences for Health, University of Milan, 20133 Milan, Italy; 3 Independent Researcher, 21100 Varese, Italy; rosamaria.fastuca@gmail.com; 4 Department of Biomedical, Surgical and Dental Sciences, University of Milan, 20122 Milan, Italy; alberto.caprioglio@unimi.it; 5 Fondazione IRCCS Cà Granda, Ospedale Maggiore Policlinico, 20122 Milan, Italy

**Keywords:** Class II malocclusion, early treatment, cephalometry, lower incisor proclination, premolar extraction, extraoral traction appliances, leeway space

## Abstract

The present study was carried out to evaluate the benefits from one-phase Class II Early Treatment (ET) using extraoral forces and functional appliances but without intermaxillary forces and eventual lower leeway space preservation compared to two-phase Class II Late Treatment (LT) with the need for extractions and full fixed appliances as well as lower incisor proclination. The ET group (*n* = 239, 115 M, 124 F, mean age 10.6 ± 1.2 years), with first premolars not in contact and the second deciduous lower molars preserved, was compared to the LT group (*n* = 288, 137 M, 151 F, mean age 12.4 ± 1.5 years). The ET group was first treated with headgears, growth guide appliances, or Teuscher activators and, in borderline crowding cases, with lower space maintenance by a lingual arch, lip bumper, or fixed utility arch. The LT group and the second phase of ET were treated with full fixed appliances including intermaxillary forces such as Class II elastics or noncompliance devices; headgear and a growth guide appliance were also used. Cephalograms and plaster models were taken before (T1) and after treatment (T2) to calculate cephalometric changes and space balance discrepancies. The differences between T1 and T2 were analyzed by a *t*-test for normally distributed data and by the Mann–Whitney Test for nonnormally distributed data at a level of *p* < 0.05. The groups were defined as statistically homogeneous at T1. A statistical analysis showed that the ET group (mean treatment time 35.3 ± 13.3 months) was significantly associated with a 22.2% lower extraction rate, 15.9% less need for a full fixed appliance, and more than 5° less incisor proclination in the nonextraction cases compared to the LT group (mean treatment time 25.9 ± 8.1 months); treatment time significantly increased in the ET group compared to the LT group. Early Class II treatment resulted in a significant treatment effort reduction in more than one third of the patients and less lower incisor proclination, even if it clinically increased treatment time.

## 1. Introduction

Class II malocclusion is certainly the most frequent malocclusion in the Western world, and it represents a percentage of about 25–30% of the malocclusions of the orthodontic population [1]. Its etiology is multifactorial, being caused by skeletal or dental factors or their combination [2]. A diagnosis of Class II typically might determine the presence of maxillary prognathism and/or mandibular retrognathism, together with a variation in the occlusion and inclination of the teeth [3]. Mandibular retrusion is a common finding, as are pronounced overjet and prominent maxillary incisors with incomplete lip closure, also with an increased risk for dental trauma and functional alterations [4].

A correct diagnosis to decide the most appropriate treatment plan should consider not only the interarch relationships but also skeletal discrepancy, age, and patient compliance. Therapeutic choices for Class II malocclusion may include extraoral appliances, functional devices, and fixed multibracket appliances with Class II elastics or noncompliance appliances [5]. Headgears are commonly used in orthodontic treatment among children with a Class II occlusion and moderate crowding; the treatment goal is to distalize or at least to stabilize the maxillary complex [6]. Similar effects with an additional slight mandibular growth stimulation are achieved by growth guide appliances [7]. Functional appliances are used to enhance mandibular growth, and different designs have been described in literature; however, all of them have the purpose of relocating the mandible in a forward position [8]. Dentoalveolar changes with noncompliance intermaxillary appliances are also widely documented [9,10]; the principal controversy concerning the mode of action of functional appliances is about their effect on mandibular growth. Finally, after the adolescent growth spurt, however, the therapeutic possibilities for severe skeletal discrepancies are limited to camouflage treatment by premolar extractions [11], molar distalization [12], or jaw repositioning by orthognathic surgery [13].

Although Class II malocclusion is largely spread, the optimal age for treatment has been debated. There are two general strategies prevailing today for the timing of treatment for Class II malocclusion; the treatment can be carried out in one or two phases. The first call for treatment may involve a first intervention (“phase I” or “early treatment”) during preadolescent years (ages 8–11) in the mixed dentition, which is sometimes followed by a second intervention (“phase II” or “late treatment”) during early adolescence (ages 12–15) in the permanent dentition. The latter approach to the timing of Class II treatment is to accomplish the entire correction during the adolescent years [14]. Early treatment is therefore defined as a first part of a comprehensive treatment, begun prior to adolescence and performed to achieve Class II dental and skeletal correction with a second phase eventually required for the completion of the orthodontic treatment.

In literature, there are several thoughts on these two different therapeutic approaches, and it remains controversial whether early treatment is effective or not, as well as the difficulty of standardizing treatment protocols between clinicians [15]. Clinicians advocating one-phase treatment argue that this approach decreases total treatment time, reduces treatment costs, and reduces the consequences of a prolonged treatment including enamel demineralization and root resorption [1]. The proponents of the two-phase treatment suggest that there are significant benefits to early intervention including the following: early normalization of the skeletal pattern and growth, easier and quicker future orthodontic treatment, reduced risk of trauma, and early improved aesthetics in some patients [1].

Independent of one-phase or two-phase interventions, different biomechanics can be applied to correct or dentally compensate skeletal discrepancies. One of the side effects of most of these biomechanics is proclination of the lower incisors. In patients with a thin labial bone plate and thin gingival biotype, incisor protrusion could lead to alveolar bone dehiscence and subsequent gingival recession [16]. The average lower incisor protrusion is reduced if a higher rate of extraction treatments is performed. It may therefore be stated that severe Class II may be more predisposed to gingival recessions due to a smaller lower apical base, greater base discrepancy, and, consequently, a greater need for dentoalveolar compensation [17]. Furthermore, a greater proclination of the incisors can be also associated with a worsening of soft tissues and profile [18].

The aim of this study was to analyze if early Class II treatment without intermaxillary forces and eventual preservation of the lower leeway space reduces lower incisor proclination and the need for extraction and full fixed appliances. The design was a retrospective study testing the null hypothesis that there was no difference in the clinical outcome between early and late Class II treatments.

## 2. Materials and Methods

A total of 527 consecutive patients attending the office of one of the authors (RM) for debonding or retention control between November 2012 and December 2018 were included in this retrospective study. The study was conducted in accordance with the Declaration of Helsinki and approved by the Ethics Committee of the University of Milan (protocol code 2020-retro-01/1, date 18 January 2020). Patients were selected according to the following inclusion criteria: mild to severe Class II molar/canine relationship (average sagittal intercuspation of canines and first molars more than one third of a premolar width); aged between 8–18 years before treatment start; good quality radiographs with adequate landmark visualization at T1 and T2. The following exclusion criteria were used to remove patients from the analysis: previous orthodontic treatment; presence of any dental anomalies in number, size, and form; presence of posterior crossbite; unilateral extractions or single lower incisor extraction; severe skeletal or dental asymmetries; orthognathic surgery performed; and patients with an obvious lack of compliance.

Based on the treatment type, 2 groups of patients were compared: Early Treatment group (ET) and Late Treatment group (LT). A total of 239 patients (115 M, 124 F, mean age 10.6 ± 1.2 years) were included in ET group, while 288 patients (137 M, 151 F, mean age 12.4 ± 1.5 years) formed the LT group. For allocation to the ET group, the first permanent premolars must not have been in occlusal contact at treatment start, and the second deciduous molars must have been preserved in case of lower crowding. Allocation to the LT group was performed if at least one permanent first premolar couple was in occlusal contact with the antagonist at start of the treatment and/or if the lower second deciduous molars had exfoliated early or had been extracted in the event of lower crowding.

Early treatment was performed using extraoral traction with headgears, growth guide appliances, or Teuscher activators [19]. No intermaxillary forces were used. The decision for extraction, nonextraction, or nonextraction attempt with later re-evaluation was individually made by the treating orthodontist (RM), roughly following the proposition by Richuse et al. [20]. According to these authors, not only the space balance, but also the vertical pattern, axial inclination of the lower incisors, the gingival phenotype, and the degree of Class II malocclusion must be taken into account for the extraction decision; however, the space balance in the mixed dentition was given more importance than in the mentioned study. In ET borderline cases (lower space balance roughly between −3 mm and +3 mm according to Tanaka [21]), a preservation of leeway space was performed by lingual arch, lip bumper, or fixed utility arch; special care was taken not to change the lower arch form nor increase the intercanine width. For the LT group and second phase of the ET group, any appliances were used including full fixed appliances with intermaxillary forces, such as Class II elastics and noncompliance devices (flexible and hybrid ones). In addition, headgears and growth guide appliances were used. During the second phase or late treatment and according to the individual diagnosis, patients were treated with either no extractions or bilateral extractions in both jaws. In some cases, only a bilateral extraction in the upper jaw was carried out with a therapeutic molar Class II and canine Class I at the end of the orthodontic therapy.

Before treatment start, an informed consent for releasing radiological diagnostic records for scientific research was obtained. Cephalograms and plaster models were taken at T1 (before treatment start) and T2 (end of active treatment).

Lower space analysis was performed on the model casts at T1. Cephalograms were traced manually and, finally, superimposed on the anterior cranial base.

The following linear and angular measurements were taken at T1 and T2: ANB, Wits, SN/MeGo, OVJ, OVB, mean sagittal intercuspation of canines and molars separately (% premolar-width Class II), total lower space balance, lower incisor inclination to the Frankfurt Horizontal (−1/FH) and to the mandibular base (−1/MeGo), and distance of the lower incisor’s labial surfaces to NPg (−1–NPg). If an intentional protrusion of retruded lower incisors was performed to regain space, these cases were excluded from the evaluation of proclination, unless the final position of the incisors was outside the first standard deviation of the normal positional values at T2. As additional outcome parameters, the dichotomous question (Yes/No) of need for a full fixed appliance (FFA) and need for extraction were analyzed. For the extraction need, three outcome options were possible: no extraction (NonEx), extraction only in the upper jaw with a “therapeutic Class II” molar occlusion (MonomaxEx), and extraction in both arches (BimaxEx). The NonEx LT group patients were further divided into two subgroups regarding the position of lower incisors at T2: subgroup A with correct lower incisor position (−1/FH ≥ 58° and −1/NPg ≤ 5.5 mm, which means less than half a standard deviation from the ideal reference in the protrusive direction for both measurements) and subgroup B with protruded lower incisors (−1/FH ≤ 52° and/or −1/NPg ≥ 6 mm, which means at least one of these measurements was lying 1.5 standard deviations or more outside of the ideal references in the protrusive direction). Finally, treatment time was also calculated for both ET and LT groups. All procedures and measurements were performed by one single well experienced operator (RM).

The method error was quantified with the method of moments variance estimator. A total of 20 lateral cephalograms were randomly selected and retraced by a second orthodontist (CA). A paired *t*-test showed no significant differences between the 2 series of cephalometry. The mean error and 95% confidence interval among the repeated data were 0.7 mm (0.5–0.8) for linear measurements and 0.8° (0.6–0.9°) for angular measurements; reliability coefficient (r) for linear and angular measurement ranged from 94% to 98% and from 92% to 97%, respectively. The nonbinary data were checked for normal distribution. Significant differences were analyzed by the *t*-test for normally distributed data and by the Mann–Whitney Test for non-normally distributed data at a level of *p* < 0.05. For the binary data (need for extraction and/or full fixed appliance), 95% confidence intervals (CI) were calculated, and the significance of the differences analyzed by the Chi-square test.

## 3. Results

The initial homogeneity of the two investigated groups ET and LT were tested as displayed in Table 1.

There were no statistically significant differences between the ET and LT parameters except for a 1.6° smaller jaw divergency (*p* < 0.001), a 0.6 mm increased overbite (*p* < 0.05), and a 1 mm reduced total lower space balance (*p* < 0.05) in the LT group. All these differences were to be expected with an ET and a LT group whose mean age was almost two years apart; furthermore, the mean age was statistically significant between the ET and LT groups, of course (*p* < 0.001). The average dental Class II occlusion was about two thirds of a premolar width in both groups with a 6% premolar width less for the molars in the LT group (*p* < 0.05). The compared groups can therefore be judged as comparable except for the inherent age-specific differences.

Table 2 summarizes the results for the extraction patterns and need for full fixed appliances for all the evaluated patients.

A data analysis showed that there were 82.4% NonEx treatments in the ET group, whereas there were only 60.2% NonEx treatments in the LT group; the difference of 22.2% between the groups was statistically significant (*p* < 0.001). The percentage of BimaxEx treatments was almost identical with 16.0% and 16.6% for the ET and LT groups, respectively. The NonEx difference was due to the much higher need for MonomaxEx in the LT group when compared to the ET group showing a statistically significant difference of 21.2% (*p* < 0.001). The inferential analysis showed a statistically significant difference of 15.9%, with 78.9% and 94.8% of FFA patients for the ET and LT groups, respectively (*p* < 0.001). The differences between the need for dental extractions only for patients who required a full fixed appliance are shown in Table 3.

A total of 77.7% of patients in the ET group did not need any extraction compared to only 59.5% of the patients from the LT group. A difference of 20.9% for the MonomaxEx ratios was shown between the two groups and was higher in the LT group. Statistically significant differences were reported for all these tested variables (*p* < 0.001). When adding up the differences for the FFA need and the difference of the NonEx ratios of the patients with FFA, it can be stated that there was a reduction of treatment effort of 34.1% in the ET group, either because there was no need for extraction and/or for a full fixed appliance.

The sagittal positional changes of the lower incisors and correlated inferential statistics between groups are reported in Table 4 and Table 5.

In NonEx patients, the lower incisors were protruded by 5.1° more for the LT group in relation to the Frankfurt Horizontal (−1/FH) and 5.5° with respect to the mandibular plane (−1/MeGo) compared to the ET group; statistical significance was observed for both measurements (*p* < 0.001). The linear sagittal position of the incisors was also statistically significant with a more anterior position of 0.8 mm (−1-NPg) in the LT than ET group (*p* < 0.05). The same parameters showed smaller differences if only patients with FFA were considered. Statistically significant differences were observed in the LT group with only 3.7° more proclination to the Frankfurt Horizontal (−1/FH) and 4.3° more to the mandibular plane (−1/MeGo) compared to the ET group (*p* < 0.001). Conversely, no statistical significance was reported regarding the sagittal position of the lower incisor labial surface (−1-NPg) that was only 0.4 mm more anterior in the LT group compared to the ET group. Table 6 shows the comparison between subgroups A and B of the NonEx LT patients.

A descriptive statistical analysis showed that the −1/FH, −1/MeGo, and −1-NPg parameters were significant between subgroups at T1, with a worse position of lower incisors in subgroup B in relation to the FH (*p* < 0.001), MeGo (*p* < 0.05), and NPg (*p* < 0.001) planes.

Finally, regarding the duration of the entire active treatment, it was shown that LT patients had an advantage in terms of a shorter therapy of 9.4 months compare to the ET group, on average. In particular, the treatment of the LT group lasted 10 months less for the NonEx, 21.3 months less for MonomaxEx, and 9.5 months less for BimaxEx subgroups compared to patients from the ET group; a statistical significance was observed for all the tested variables between the two groups (*p* < 0.001). On the other hand, the treatment duration with the FFA was 2.7 months shorter in the ET group when compared to the LT group (*p* < 0.001). Specific durations between treatment types and groups are summarized in Table 7.

## 4. Discussion

The goal of early treatment is to reduce the severity of an existing problem, to keep it from worsening, or to reduce the complexity of future treatment. The recommendation for early treatment, however, is most frequently based on empirical or clinical judgement. The limited scientific evidence may be due to the definition of “early treatment”, which includes different periods from primary, through early and late mixed dentition; including such a large period can lead to different results. Furthermore, many published reports present different inclusion criteria as well as different outcome variables. Actually, there is very little literature available regarding the tested outcomes of the present study, and the results are controversial. According to a review by Brierley et al., the evidence suggests that routine early treatment is not effective at improving final orthodontic outcomes, which does not mean, however, that early treatment is never indicated [1]. A recent systematic review was performed to compare one- and two-phase treatments for Class II malocclusion [15]; from the major studies on the topic, it is possible to assume that both one-phase and two-phase treatments are effective in correcting Class II malocclusions, with no significant difference for any outcome, except new incidences of incisor trauma, which was significantly less for the early treatment group. In particular, the North Carolina group described the effects of one-phase versus two-phase treatment; the authors did not find any advantage in terms of the number of extractions and the need for orthognathic surgery: the number of children who underwent extractions, in fact, showed no statistically significant outcome differences between the early and late treatment groups [22,23,24]. It must be realized though, that the North Carolina study had major patient dropouts, used a functional appliance with intermaxillary forces (Bionator), and excluded patients who did not receive a second phase of treatment with fixed appliances. However, it is exactly this group that made a major contribution to the differences found in the present investigation. Nonetheless, the previously mentioned study from North Carolina found less lower incisor proclination in the ET group, which conforms with the findings in the present study. In addition, O’Brien et al. from Manchester University investigated the differences between early and late treatment regarding overjet, skeletal pattern, and extraction rate. The authors reported no statistically significant differences between the groups for the investigated parameters, but, unfortunately, they did not consider lower incisor proclination [25]. However, the study excluded early treatment patients who did not receive a second phase of fixed orthodontic treatment. In relation to the effect of the Class II treatment on the lower incisors, Vasilakou et al. reported a negative proclination of the mandibular incisors as a side effect of intermaxillary mechanics used to advance the mandible in the early treatment for Class II malocclusion [26]. However, such intermaxillary forces were avoided in the ET group of the present study; the functional appliance used was the Teuscher activator, which does not cause lower incisor proclination [19]. In contrast to all these reports, Florida University found beneficial effects of early Class II treatment [27,28]. Although the dental effects relapsed, they proved the skeletal ones to be stable [27]. The present study clearly contradicts the scientific evidence found so far, except for the evidence from Florida University. This may be attributed to different definitions of **“**early treatment**”**, different inclusion criteria or outcome variables, and/or better patient compliance/management in the present study.

Full fixed appliances were required in 15.9% fewer of the patients of the ET group compared to the LT group. The major portion of these treatments without the need for a full fixed appliance were NonEx treatments. By excluding the patients without full fixed appliance, the extraction ratios of the patients who needed a full fixed appliance could be assessed. In the present study, we observed that the difference of the NonEx ratios between the ET and LT groups was only 18.2% in favor of the ET group. By adding up the two percentages, it can be stated that the early treatment of Class II resulted in a significantly reduced treatment effort in more than one third of the patients (34.1%) due to the fact that either no extraction had to be performed and/or because no costly full fixed appliance was needed. In the North Carolina, Florida, and Manchester studies, early treatment patients who did not receive a full fixed appliance either dropped out or were excluded. The present study is the first one to investigate the percentage of these patients and incorporate their results in the treatment outcome variables. The incorporation of these patients may have been the major contribution to the differences to other studies on the benefits of early Class II treatment. Additionally, most of the other studies did not incorporate lower incisor proclination as an outcome variable, which might also have influenced the extraction pattern.

Using lower space maintenance is an effective procedure to block the leeway space for the sake of incisor crowding resolution during late mixed dentition in borderline crowding patients [29]. If a Class I molar relationship is achieved without the use of intermaxillary forces in such cases, lower incisor proclination can be avoided. It might be assumed, therefore, that the use of lower leeway space maintenance may reduce the need for lower premolar extraction.

Although statistical significance was observed, there was no clinically relevant differences concerning the BimaxEx ratios in this study. It can be stated that a patient who absolutely needs bimaxillary extractions cannot be turned into a NonEx-patient just by an early treatment start. The main differences for the NonEx ratios were based on the fact that MonomaxEx treatments needed to be performed in the LT group about 12 times more often than in the ET group. This could, of course, be compensated for by upper arch distalization with the aid of skeletal anchorage instead [30]. However, this is a very costly alternative [31,32].

One of the main outcomes of the present study was the inclination control of the lower incisor. It is obvious that, on average, less incisor proclination will result in more patients receiving extraction treatment. Nevertheless, it is hard to prove the benefits of early treatment without simultaneously looking at both factors: extraction pattern and lower incisor proclination [33]. If a higher ratio of extractions is performed, and, consequently, there is less proclination in one group when compared to the other (or vice versa), no conclusion can be drawn. However, in the present study, there was more than a 5° less proclination of the NonEx ET patients, although 22.2% fewer of these patients received extraction treatment in comparable initial situations, thus indicating a clear and significative effect of the early intervention. In order to guarantee realistic data of the incisor proclination, maintaining the lower arch form and intercanine width was a principal intent during all the performed treatments [34]. The difference of incisor proclination between the ET and LT groups was smaller when the patients without full fixed appliance were excluded. This leads us to suspect that the full fixed appliance has a major impact on lower incisor proclination despite a doubtful periodontal side effect in accordance with previous studies [35,36]. Three possible mechanisms can be assumed, which need further investigation related to the importance of an early approach to Class II malocclusion: crowding resolution should read as intentional incisors proclination, intermaxillary Class II biomechanics, and lower incisor intrusion causing a labial tipping due to the labially eccentric force application. It is well known that full fixed appliances produce incisor proclination due to the necessity of crowding resolution [37]. Furthermore, despite being effective in correcting Class II, intermaxillary elastics produce dentoalveolar side effects, such as mandibular incisor proclination and their intrusion [38].

For the clinician, it is often difficult to decide which Class II treatments have to be performed early and which can be postponed until permanent dentition. A significant number of patients need early treatment anyways for some local factors, such as lateral crossbite with a need for maxillary expansion, impacted canines, attrition of incisors due to deep overbite, transpositions, or major skeletal deviations. In order to evaluate which Class II treatments without such local factors can be postponed from mixed to permanent dentition without the danger of overproclination and higher percentages of extraction, the initial parameters of the two subgroups of only the LT NonEx patients were also compared; the post-treatment inclination of the lower incisors to FH and NPg was used to classify patients into subgroups A (correct incisor position at T2) and B (protruded lower incisors at T2). The significant initial differences between subgroups A and B were related to skeletal Class II, skeletal divergency, and lower incisor positioning. The other investigated parameters were not significant. However, space balance requires special attention: the mean lower space balance of the LT NonEx subgroup B was positive by 1 mm. Nevertheless, subgroup B resulted in an overproclination of the lower incisors. Taking into account that there is a natural reduction of the space balance when the first molars take the leeway space, it could be argued that there needs to be a significantly positive space balance in the mixed dentition in order to be sure not to overprocline the lower incisors if a LT NonEx Class II treatment approach should be chosen. According to the results obtained, the authors would like to recommend a guideline for Class II patients with delayed treatment that starts in the permanent dentition: only minor skeletal Class II, hypodivergency, retruded lower incisors, and excessive space balance (≥5 mm) in early mixed dentition. These features were shown by about only 15% of the ET group.

The price to pay for the advantages of an early treatment start is a prolonged overall treatment time by 9 months on average. According to previous studies on early treatment efficiency, a treatment starting at the mixed dentition age simply increased the number of patient attendances and the duration of treatment [1,25]. In our study, special attention was on the huge difference of 21 months for the total treatment time in the MonomaxEx ET vs. the LT group. The LT patients were planned from the very beginning as were the MonomaxEx treatments in most of the cases. The ET MonomaxEx patients were only 5 out of 239 and were started as NonEx treatment attempts. The concept was only later shifted to MonomaxEx due to insufficient sagittal correction; this resulted in a dramatically prolonged overall treatment time. For bimaxillary extraction cases, a systematic review showed that early serial extraction resulted in a significantly shorter active fixed appliance treatment time, although the total treatment time was prolonged in comparison with a late treatment start [39]; this is not astonishing as an early and spontaneous crowding resolution makes orthodontic treatment easier in stage two. It seems logical that if early treatment may reduce the complexity of later treatment, there should be a reduction in phase two treatment time. In our study though, the difference of FFA treatment time between the two groups was less than 3 months, whereas the total treatment time was increased by about 9 months in the ET bimaxillary extraction group. This may be attributed to the fact that all patients in this study were Class II patients, and that the first treatment phase was more addressed to Class II correction than to crowding resolution. In general, it may be concluded that with the progress of dental development, the number of appointments decreases [40].

Finally, individualized treatment planning cannot be randomized. Therefore, the principal question of the present study cannot be investigated in a randomized controlled trial; in order to minimize any potential bias, it used retrospective data from a large consecutive and prospective instead. One potential limitation may be the fact that not all patients in the ET group received lower leeway space maintenance; excluding these patients would have resulted in a serious selection bias and had a major impact on the average space balance in the ET group. After all, lower leeway space defense is an important component of early Class II treatment. Furthermore, all rapid maxillary expansion patients and patients with unilateral extractions a priori were excluded in this study. In fact, the majority of the expansion cases were early NonEx patients, whereas unilateral extractions were more often performed in the permanent dentition. It can thus be postulated that the differences concerning extraction pattern and incisor proclination are even bigger in reality, and this may require further studies to compare data with.

## 5. Conclusions

This retrospective study was carried out to evaluate the possible benefits of early Class II treatment in terms of the need for extraction and full fixed appliances as well as the amount of lower incisor proclination in nonextraction treatments. Based on the statistical analysis performed on a cohort of 527 patients, it can be stated that early treatment improved the clinical outcomes by decreasing the need for full fixed appliance therapy and extractions and also limited the proclination of the lower incisors. The null hypothesis was therefore rejected.

- Early Class II treatment without mesializing forces on the lower dentition is effective, as it reduces the treatment efforts by a lower extraction rate and full fixed appliance need in more than one third of the patients.
- Despite a decreased extraction rate, such early Class II treatment results in less lower incisor proclination.
- Early Class II treatment increases the overall treatment time but slightly reduces the full fixed appliance time if such is necessary, except for maxillary camouflage extractions after unsuccessful phase one treatment, which increases treatment time dramatically.
- The fixed appliance seems to be a contributing factor to lower incisor proclination independent of early or late treatment.
- Early Class II correction is advised as a standard protocol except for few carefully selected cases, characterized by retruded positioned lower incisors and positive space balance, in which it can be postponed until permanent dentition.

## Figures and Tables

**Table 1:** Descriptive parameters at T1.

	ET Group		LT Group			
	Mean	SD	Mean	SD	Δ	*p*
ANB (°)	4.3	1.9	4.1	2.4	−0.2	0.310
Wits (mm)	1.8	2.4	2.2	2.6	0.4	0.099
SN/MeGo (°)	27.0	4.5	25.4	4.4	−1.6	<0.001
OVB (mm)	4.3	1.0	4.9	1.5	0.6	<0.05
OVJ (mm)	5.5	2.3	5.0	2.1	−0.5	<0.05
−1/FH (°)	62.1	6.5	63.0	6.4	0.9	0.122
−1/MeGo (°)	94.3	6.5	94.3	8.6	0.0	0.871
−1-NPg (mm)	3.7	2.6	3.5	2.7	−0.2	0.435
Lower space balance (mm)	1.9	3.7	0.9	3.6	−1.0	<0.05
Intercuspation 6 ± 6 (%)	74	26	68	25	−6	<0.05
Intercuspation 3 ± 3 (%)	62	26	64	22	2	0.457

**Table 2:** Percentage of extraction and full fixed appliance (FFA) need.

	ET Group		LT Group			
	Mean	95% CI	Mean	95% CI	Δ	*p*
NonEx (%)	82.4	77.4–87.3	60.2	54.4–65.9	−22.2	<0.001
MonomaxEx (%)	1.7	0.0–3.7	22.9	18.2–28.1	21.2	<0.001
BimaxEx (%)	16.0	11.2–20.7	16.6	12.2–21.0	0.6	<0.001
FFA (%)	78.9	73.7–84.3	94.8	92.1–97.5	15.9	<0.001

**Table 3:** Percentage of extraction need for full fixed appliance (FFA) patients.

	ET Group		LT Group			
	Mean	95% CI	Mean	95% CI	Δ	*p*
NonEx (%)	77.7	71.6–83.7	59.5	53.6–65.4	−18.2	<0.001
MonomaxEx (%)	2.2	0.0–4.6	23.0	17.9–28.1	20.9	<0.001
BimaxEx (%)	20.2	14.4–26.1	17.5	12.9–22.1	−2.7	<0.001

**Table 4:** Lower incisors changes in NonEx Patients (intentional proclination for space regain excluded).

	ET Group		LT Group			
	Mean	SD	Mean	SD	Δ	*p*
−1/FH (°)	−1.7	6.0	−6.8	7.0	−5.1	<0.001
−1/MeGo (°)	2.1	6.0	7.6	7.2	5.5	<0.001
−1-NPg (mm)	−0.1	1.7	0.7	1.9	0.8	<0.05

**Table 5:** Lower incisors changes in NonEx Patients with FFA need (intentional proclination for space regain excluded).

	ET Group		LT Group			
	Mean	SD	Mean	SD	Δ	*p*
−1/FH (°)	−3.6	5.8	−7.3	7.0	−3.7	<0.001
−1/MeGo (°)	3.9	6.0	8.2	3.9	4.3	<0.001
−1-NPg (mm)	0.3	1.9	0.7	1.9	0.4	0.101

**Table 6:** Comparison of descriptive parameters at T1 of Subgroups A (*N* = 61; selection parameters at T2: −1/FH ≥ 58° and −1/NPg ≤ 5.5 mm) and B (*N* = 18; selection parameters at T2: −1/FH ≤ 52° and −1/NPg ≥ 6 mm) of patients from NonEx LT group.

	Subgroup A		Subgroup B			
	Mean	SD	Mean	SD	Δ	*p*
ANB (°)	3.3	1.8	4.6	1.7	1.3	<0.05
Wits (mm)	1.6	2.3	1.8	2.9	0.2	0.714
SN/MeGo (°)	24.2	4.0	26.6	3.5	2.4	<0.05
Overbite (mm)	5.0	1.2	4.8	1.3	−0.2	0.486
Overjet (mm)	4.3	1.8	4.7	1.7	0.4	0.466
−1/FH (°)	66.9	6.2	60.4	5.8	−6.5	<0.001
−1/MeGo (°)	92.1	6.3	95.5	5.4	3.4	<0.05
−1–NPg (mm)	2.1	2.3	4.9	2.1	2.8	<0.001
Lower Space Balance (mm)	2.1	2.6	1.0	1.8	−1.1	0.095
Intercuspation 6 ± 6 (%)	63	24	63	21	0.0	0.990
Intercuspation 3 ± 3 (%)	60	23	59	19	−1.0	0.901

**Table 7:** Duration in months (Mos) of active treatment.

	ET Group		LT Group			
	Mean	SD	Mean	SD	Δ	*p*
All patients	35.3	13.3	25.9	8.1	−9.4	<0.001
NonEx	34.5	13.1	24.5	8.0	−10.0	<0.001
MonoMax	48.0	10.8	26.7	8.2	−21.3	<0.001
BimaxEx	38.4	13.4	28.9	7.9	−9.5	<0.001
FFA	15.1	4.9	17.8	7.0	2.7	<0.001

## Data Availability

The data presented in this study are available on request from the corresponding author. The data are not publicly available due to privacy limitations.
